# Feasibility and adherence to moderate intensity cardiovascular fitness training following stroke: a pilot randomized controlled trial

**DOI:** 10.1186/s12883-021-02052-8

**Published:** 2021-03-22

**Authors:** Hanna Reynolds, Sarah Steinfort, Jane Tillyard, Sarah Ellis, Alan Hayes, Erik D. Hanson, Tissa Wijeratne, Elizabeth H. Skinner

**Affiliations:** 1 Department of Physiotherapy, Western Health, 176 Furlong Rd, St Albans, 3021 Australia; 2 Australian Institute of Musculoskeletal Science, Melbourne, Victoria Australia; 3 The Institute for Health and Sport, Victoria University, Melbourne, Australia; 4 The University of Melbourne, Melbourne, Australia; 5 University of North Carolina, Chapel Hill, NC USA; 6 Department of Neurology, Western Health, Melbourne, Australia; 7 Monash University, Melbourne, Victoria Australia

**Keywords:** Stroke, Aerobic exercise, Physical fitness, Mood, Randomized controlled trial

## Abstract

**Background:**

Stroke is a leading cause of disability worldwide and the cardiovascular fitness levels of stroke survivors are diminished to an extent that impairs functioning and activities of daily living performance. While cardiovascular training seems an empirically appropriate intervention, the optimal dosage and intensity of cardiovascular training in stroke survivors remains unclear. The aim was to determine the safety and feasibility of moderate-intensity cardiovascular training following stroke, including measurement of adherence to training.

**Methods:**

A pilot, prospective, patient- and assessor-blinded randomised controlled trial conducted in a tertiary, metropolitan hospital-based community rehabilitation centre. Eligibility criteria included ambulant (> 100 m), 6 weeks-12 months post stroke. Moderate-intensity fitness training or control (low-intensity) exercise was offered biweekly for 12 weeks. Outcome measures included adverse events, peak oxygen uptake (VO_2_), functional exercise capacity (6-Minute Walk Test, 10-m Walk Test) and health-related quality of life (Short Form-36) and mood (Patient Health Questionnaire, PHQ9).

**Results:**

*Feasibility:* Seventy-one (50%) of 141 screened participants were eligible (29% did not agree to participate). Twenty participants (10 intervention, 10 control) were recruited. The median (%; IQR) supervised sessions was 19.5 (81%; 12, 20); and 20 (83%; 19, 22) in the intervention and control groups, respectively. Progression of duration and intensity was limited; mean of 10 sessions to achieve target duration (30 min). There were no adverse events. Baseline peak oxygen uptake (VO_2_) levels were low (15.94 ml/kg/min). Significant improvements in VO_2_ peak in both groups were observed (*p* < 0.05). Although there were no significant between-group differences, this feasibility trial was not powered to detect change.

**Conclusions:**

Moderate-intensity fitness training was safe but achievement of target duration and intensity was challenging for stroke survivors. A definitive adequately-powered randomised trial is required. Alternative fitness training protocols may need to be explored.

**Trial registration:**

The trial protocol was prospectively registered on the Australian New Zealand Clinical Trials Registry (ACTRN 12613000822785) on 25/07/2013.

**Supplementary Information:**

The online version contains supplementary material available at 10.1186/s12883-021-02052-8.

## Background

Stroke is a leading cause of disability [1, 2] with more than half of stroke survivors requiring assistance with daily living activities [3] and one-third affected by post-stroke depression [4]. Sedentary behaviour is highly prevalent (> 77%) in people after stroke [1]. While physical inactivity is an independent risk factor for stroke [5], moderate-intensity exercise may have a protective effect against subsequent stroke and vascular events [6].

Cardiorespiratory fitness, measured as peak oxygen uptake (VO_2_ peak), has been estimated at 8 to 22 ml.kg.min following stroke; between 26 and 87% of age and gender-matched normative levels [7–9]. Such values fall below or just surpass the minimum aerobic capacity required for independent living (15 and 18 mL.kg.min for women, and men, respectively) [8, 10]. The presence of very low cardiorespiratory fitness following stroke is likely to contribute to disability and dependence.

Meta-analyses demonstrate cardiorespiratory training improves fitness, walking speed and endurance in stroke survivors [7, 11, 12], whilst efficacy on mood and quality of life (QOL) has not been established [12]. International guidelines for the general and post-stroke population recommend 30 min of moderate-intensity aerobic activity (40–59% heart rate reserve) on most days of the week [13–15]. However, a recent Cochrane review found an ‘optimal dosage for the content of training for people with stroke has yet to be established’ [12]. Actual dosages achieved by stroke survivors (rather than dosage prescribed) has been under-reported in previous studies [7, 12] which may limit analysis of dose-response [16].

This pilot study aimed to inform the design of a future adequately-powered randomized controlled trial (RCT), with the following specific objectives: 1) evaluate the feasibility of recruitment and trial procedures; 2) evaluate the safety and feasibility of implementation of the recommended CV training dosage, including actual achievement of target dosage in clinical practice, in addition to usual care, and 3) estimate preliminary effect of moderate-intensity CV training, compared to a low intensity, on CV fitness (defined as VO_2_ peak), walking speed and endurance, QOL and depression.

## Methods

### Trial design, setting and location

This study was an investigator-initiated, pragmatic, patient- and assessor-blinded, parallel group pilot randomised controlled trial. The study was conducted in a community-based rehabilitation (CBR) service of a tertiary, metropolitan hospital (Sunshine Hospital, Melbourne, Australia), during the period October 2013 to May 2016, with follow-up completed in August 2016.

### Ethics and consent statement

The institutional review board approved the study (Melbourne Health HREC Project number: 2013.105). The trial protocol was prospectively registered on the Australian New Zealand Clinical Trials Registry (ACTRN 12613000822785) and reported according to the Consolidated Standards of Reporting Trials (CONSORT) statement and extensions for a pragmatic non-pharmacological intervention trial [17, 18], and the TIDieR checklist [19], with pilot study design guidance obtained from previous publications [20, 21].

Written informed consent was provided by all participants. Informed consent was sought using accredited interpreters for participants with a non-English speaking background.

### Participants

A convenience sample of 20 participants was recruited. Eligible participants were aged 18 years and over; diagnosed with stroke (ischaemic or haemorrhagic) within the past 6 weeks (minimum) to 12 months (maximum) and able to walk at least 100 m (with or without aids or standby supervision). Key exclusion criteria (for full list, see Supplemental File 1) included pregnancy; documented medical restrictions to CV training; unstable cardiovascular, metabolic and renal co-morbidities and inability to physically participate in a cycle ergometry test (safely mount and cycle stationary exercise bike at 50 rpm (RPM)).

### Procedure

Physiotherapists screened all people with stroke attending CBR for eligibility. Potential participants underwent medical screening against the inclusion/exclusion criteria with a neurology physician prior to inclusion and randomisation. Patients underwent a battery of initial outcome measures in the week following recruitment (prior to randomization), and then attended the relevant 12-week protocol at CBR depending on randomization. Outcome measures were repeated after the 12-week training program.

### Interventions

Table 1 outlines the intervention and control protocols.

**Table 1:** Description of intervention and usual care groups

	Progressive moderate-intensity CV training	Low intensity ‘standard care’ exercise
Frequency	2/week Clinic-based supervised sessions 3/week home exercise program	2/week Clinic-based supervised sessions 3/week home exercise program
Training duration	Target duration: 30 min Commenced at 5 to 10 min. Progressed each session (as tolerated) until 30 min. (10 min bouts with rest breaks allowed as required)	Weeks 1–4: 10 min duration Weeks 5–8: 20 min Weeks 9–12: 30 min (actual exercise time, not including rest breaks and set-up)
Training intensity	Moderate intensity (40–59% HRR) calculated each session using Karvonen method: *Target Heart Rate = (Heart Rate Reserve x %intensity (VO*_*2*_*)) + Heart Rate rest* Intensity commenced at 40% HRR IF target duration achieved, intensity progressed by 5% increments as tolerated. BORG-RPE 11–13.	Low intensity < 40% HRR BORG RPE < 11 40% HRR calculated each session using Karvonen formula
Program length	12 weeks	12 weeks
Training mode	Upright stationary cycle ergometer; recumbent bike; treadmill; upper limb ergometer; stepper; cross-trainer; stairs.	Upright stationary cycle ergometer (maximum 5 min per session), walking in rails or gym, standing balance, basic strengthening (slow squats, seated quadriceps extension) and bed-based exercises.
Delivery mode	Face to face individual or small group session (maximum 3 participants)	Face to face individual or small group session (maximum 3 participants)
Location	Physiotherapy gym in a community rehabilitation service (outpatient) of tertiary metropolitan hospital	Physiotherapy gym in a community rehabilitation service (outpatient) of tertiary metropolitan hospital
Home exercise program	3 sessions/week once safe to self-monitor Parameters matched supervised sessions	3 sessions/week once safe to self-monitor Parameters matched supervised sessions

*CV* Cardiovascular, *BORG-RPE* Borg Rate of Perceived Exertion, *VO*_*2*_ Oxygen consumption

The intervention was a 12-week progressive fitness training program designed according to ACSM guidelines [13, 22], with participants aiming to progress to a total of five 30-min sessions per week of moderate-intensity (40–59% heart rate reserve) CV exercise. Individualised target heart rate (THR) was calculated each session using the Karvonen method, reflecting the rate of energy expenditure during physical activity more accurately than other prescription methods [13]. See Supplemental File 2 for additional intervention progression detail. Physiotherapists with at least 2 years’ experience with a neurology population were trained to deliver the standardised CV fitness training intervention. Attendance (or reason for non-attendance), duration achieved, adherence to target heart rate/intensity and adverse events were recorded.

The control protocol was a low-intensity ‘conventional exercise program’; designed to mimic standard stroke rehabilitation, previously shown to be conducted at low intensity (< 40% heart rate reserve (HRR)) [23, 24], and aimed to match total contact time in both groups with duration progressed from weeks 1–12. Five minutes (maximum) of low intensity exercise on a stationary bike was included to limit a cycle familiarisation effect on testing for the intervention group. Exercise selection was based on individual patient’s functional capacity. Heart rate and BORG-RPE were monitored every 5 min to ensure participants maintained low intensity exercise (calculated each session with Karvonen method). If HR exceeded low intensity, a seated rest was taken for heart rate to return below 40% HRR. Physiotherapists or allied health assistants delivered the control protocol.

Both groups were prescribed a home exercise program (HEP, see Supplemental File 3), incorporated to reduce reliance on clinical resources, where two centre-based sessions were deemed to be clinically feasible based on usual capacity of community rehabilitation service resources. Participants in both groups continued usual care rehabilitation (i.e. physiotherapy, excluding fitness training, and multidisciplinary allied health) at the discretion of the treating clinicians. No modification of interventions occurred during course of study.

### Randomisation

The randomisation sequence was computer generated by a research team member (who did not participate in group assignment of enrolled participants), using a random numbers table in Microsoft Excel with a 1:1 allocation ratio, to the intervention or standard care (control) group. The participant allocation sequence was sealed in individual, consecutively numbered opaque envelopes. The investigators randomised participants following baseline assessment by opening the envelopes in sequential order according to recruitment date. Randomisation was stratified by beta blocker use (Yes/No), to ensure equal numbers of patients in both groups, to avoid skewing results based on beta-blocker status.

### Blinding

Participants were informed that they would receive one of two different exercise interventions with the intent to blind participants to group allocation. Intervention and control group participants were not present in the gym at the same time. The neurology medical team (who assessed for medical suitability and stroke severity), outcome assessors and statisticians were blinded to group assignment. It was not possible to blind therapists who provided the intervention or control protocol.

### Demographic descriptive data

Baseline demographic data were collected including age, gender, time since stroke, type of stroke (ischaemic or haemorrhagic), and beta-blocker usage. Stroke severity was assessed by a blinded neurology physician using the National Institutes of Health Stroke Scale (NIHSS) and modified Rankin scale (mRS). Both scales are reliable and valid measures in stroke survivors [25, 26]. Accredited interpreters were used for participants with a non-English speaking background to conduct outcome measurement and intervention.

### Feasibility, safety & adverse events

Feasibility was assessed by reporting eligibility and consent rates; the proportion of outcome measure completion, attendance, retention, and adherence to training protocols. Safety during outcome measurement or intervention/control protocols was assessed by the occurrence of any adverse events, which included falls, cardiac, respiratory, or new neurological abnormalities, and new musculoskeletal pain.

### Clinical outcome measurement

The following outcomes were measured at baseline and following completion of the 12-week intervention:

### Cardiorespiratory Fitness (VO_2_ Peak) - Graded Exercise Test (GXT)

Measurement of maximum oxygen uptake (VO_2_max) is the gold standard for determining cardiorespiratory fitness [13]. However, in clinical populations (including stroke), peak oxygen update (VO_2_ peak, the highest VO_2_ attained during incremental exercise before symptoms or safety criteria result in termination of testing) is preferred [8, 27].

A graded exercise test (GXT) was conducted to determine VO_2_ peak, according to ACSM guidelines [13], on an electronically braked upright cycle (Monark) ergometer with a 12 lead electrocardiogram (ECG; Mortara) continuously monitoring heart rate (see Supplemental File 4).

### Functional exercise capacity (6MWT, 10MWT)

The Six-Minute Walk Test (6MWT) is a reliable and valid measure of functional exercise capacity and walking ability following stroke, with a minimum detectable change of 54.1 m [28]. Testing was conducted with standardised instructions [29]. The distance walked (metres) within 6-min was recorded. The 10-m walk test (10MWT) has excellent test-retest reliability (ICC =0.95 to 0.99) and validity in the stroke population [30] and was used to assess fast and self-selected walking speed. Testing was conducted with standardised instructions and time taken to walk the middle 10 m of a 14 m walkway was recorded. The best result of three tests was taken and converted to velocity (metres per second).

### Health-related quality of life and mood

Health-related quality of life was assessed with the Short Form-36 (SF-36), Australian version, Version 2 [31, 32], and mood was assessed using the English-language version of the Patient Health Questionnaire (PHQ-9), a 9-item scale (total scores range from 0 to 27), recommended as a screening tool for post stroke depression [4], see Supplemental File 5.

### Statistical analysis

Feasibility, adverse events and outcome measure completion rates were calculated using simple count and proportion data. Statistical analysis was performed by a statistician blinded to group assignment using SPSS Statistics™ Version 22.0.0.0 (IBM Corporation, Armonk, NY, USA). All data are presented as median (IQR) or mean (SD) unless otherwise specified. Normality of distributions was assessed using the Kolmogorov-Smirnov statistic. Missing data were ignored and no data were imputed. As this was a pilot trial, no interim analyses were planned, and no stopping guidelines were implemented based on prior existence of safety data.

Where appropriate according to distribution, independent and paired *t-*tests were used to analyze parametric differences over time between and within group respectively and non-normally distributed data were analyzed using the Mann-Whitney U for independent samples and Wilcoxon-signed ranks tests for dependent group data. *p* < 0.05 was accepted as statistical significance. Cohen’s effect size was calculated and interpreted within-group from baseline to follow-up; and between-group on change scores (interpreted as 0.4 or less = small; 0.5 = moderate and 0.8 = large) [33]. Estimated sample sizes for a future adequately powered RCT design were estimated using G*Power (Version 3.1.9.2, Universität Düsseldorf, Düsseldorf, Germany) [34].

## Results

### Screening, eligibility and recruitment rates

**Fig. 1:**
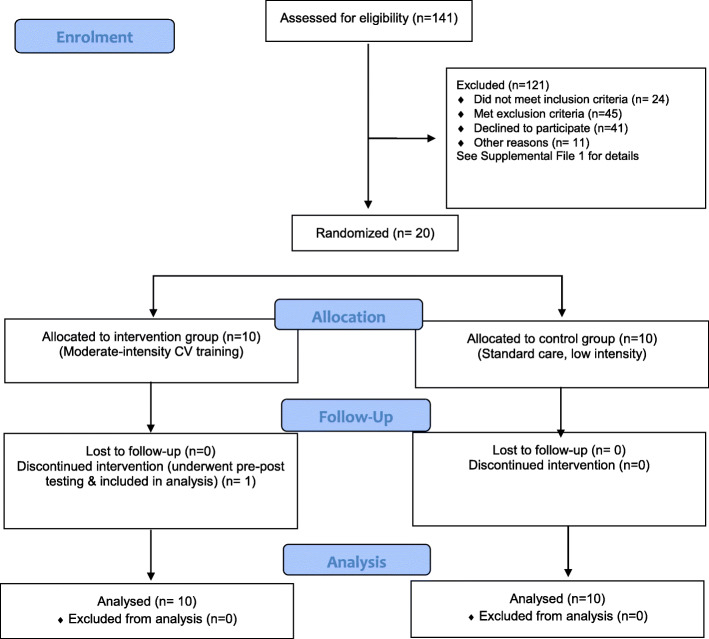
CONSORT Diagram

One-hundred and forty-one patients were screened to recruit 20 participants (Fig. 1). Sixty-nine (49%) were excluded on criteria, and 41 (29%) did not consent to participation.

Five patients who consented were excluded: two were not medically cleared, and three withdrew prior to randomisation (see Table 2 for full list of reasons for exclusion).

**Table 2:** List of reasons for exclusion

Reason	n
**Not meeting inclusion criteria**	**24**
Inability to walk 100 m	20
> 12 months post stroke	4
**Met exclusion criteria**	**45**
Cardiovascular or pulmonary contraindications	26
Insufficient expressive and receptive communication	8
Inadequate physical capacity to complete testing ie. unable to mount upright bike	3
Severe renal impairment; end stage renal failure; kidney transplant	2
Documented medical restrictions	2
Uncontrolled diabetes	1
Insufficient cognition for consent	1
Other	2
**Declined**	**41**
Inability to commit to attend 2/week (work; family; rehab; transport)	15
Not agreeable to participation in fitness training or research	17
Not stated	9
**Other**	**11**
Not given medical clearance	2
Missed (not screened)	6
Consented to participate then withdrew prior to randomisation *Moved interstate (1), returned to work (1), new onset knee pain (1)*	3

### Participant demographics

Demographic and other characteristic data are summarized in Table 3. Eighteen (90%) of recruited participants were male, and all four patients with haemorrhagic stroke who were recruited were randomised only to the intervention group. Baseline stroke severity assessed using the NIHSS showed six participants with moderately severe impairment (NIHSS 5–14) and 13 with mild impairment (NIHSS < 5) [*n* = 1 missed]. Most participants were in the sub-acute phase post stroke, with 16 (80%) recruited within 6 months; nine (45%) of these within 3 months following stroke. The intervention group were recruited significantly earlier (*p* = 0.02) and had a higher proportion of left sided lesions (*p* = 0.03). There were no other statistically significant differences in baseline demographics between groups.

**Table 3:** Demographic and clinical details of the cohort. Data are mean (SD) unless otherwise specified

	Cohort (***n*** = 20)	Intervention (*n* = 10)	Control (*n* = 10)
Age	57.5 (11.2)	54.6 (8.9)	60.3 (12.9)
Male, n (%)	18 (90)	10 (100)	8 (80)
Diagnosis			
- Ischaemic stroke, n (%)	16 (80)	6 (60)	10 (100)
- Haemorrhagic stroke, n (%)	4 (20)	4 (40)	0 (0)
Stroke location			
- Right-side lesion n (%)	10 (50)	2 (20)	8 (80)
NIHSS, median (IQR)^a^	3.0 (2–5)	3.5 (2–5)	2.5 (1–4)
mRS, median (IQR)	2.0 (1–2)	2.0 (1–2)	2.0 (1–3)
Beta-blocker usage, n (%)	2 (10)	0 (0)	2 (20)
Diabetes, n (%)	4 (20)	2 (20)	2 (20)
Time to recruitment (days), median (IQR)			
- From stroke date	109.5 (72–175)	88.0 (65–155)	128.5 (88–225)
- From CBR admission	41.5 (21–89)	48.5 (17–86)	41.0 (22–119)
Trial session attendance, mean (SD)	19 (3.6)	17.9 (4.4)	20 (2.5)
Interpreter required, n (%)	5 (25)	3 (30)	2 (20)

*NIHSS* National Institutes of Health Stroke Scale, *mRS* Modified Rankin Scale, *CBR* Community-based rehabilitation

^a^*n* = 9 (Control) for NIHSS

There were no statistically significant differences in baseline performance measures (Table 4), although the intervention group had a trend to higher, but non-significant, baseline VO_2_ peak (17.5 ml/kg/min (2.9)) compared with control (14.4 ml/kg/min (3.7), *p* = 0.06) (Table 4). Using the cut-off score of ≥10 on the PHQ-9, three (30%) participants in each group had a score suggestive of depressive illness at baseline.

**Table 4:** Pre and post data, mean (SD) unless otherwise specified

	Baseline Intervention (***n*** = 10)	Follow-up intervention	MD (95% CI) (***n*** = 10^**a**^)	***P***	Baseline control (***n*** = 10)	Follow-up control	MD (95% CI) (***n*** = 9)	***P***
VO_2_ peak	17.5 (2.9)	20.1 (4.9)	2.6 (0.5, 4.6)	0.02*	14.4 (3.7)	17.4 (4.4)	3.0 (1.0, 5.0)	0.01*
6MWT	400.9 (111.6)	443.4 (126.4)	42.5 (−10.9, 95.9)	0.11	344.8 (150.6)	370.9 (126.0)	26.1 (−15.3, 67.5)	0.18
10MWT Self	1.16 (0.29)	1.20 (0.21)	0.04 (−0.11, 0.20)	0.53	0.95 (0.38)	0.97 (0.36)	0.02 (−0.13, 0.17)	0.74
10MWT Fast	1.47 (0.36)	1.57 (0.41)	0.10 (−0.13, 0.32)	0.35	1.20 (0.47)	1.31 (0.52)	0.10 (−0.04, 0.25)	0.13
SF-36 PCS^b^	43.12 (8.65)	45.78 (8.97)	2.67 (−2.7, 8.0)	0.28	37.92 (10.16)	40.43 (9.90)	2.51 (−6.1, 11.1)	0.52
SF-36 MCS^b^	42.63 (14.72)	48.48 (17.65)	5.85 (−1.9, 13.6)	0.12	34.68 (20.35)	46.19 (10.50)	11.51 (0.09, 22.9)	0.049*
PHQ-9	7.0 (7.1)	4.2 (7.1)	−2.8 (−8.1, 2.5)	0.26	8.1 (6.2)	3.8 (3.6)	−4.3 (−6.6, −2.0)	0.003*

*CI* Confidence interval. *p* values are calculated using paired samples *t-*tests with the exception of the 10MWT analyses which were performed using the Wilcoxon-signed ranks test. VO_2_ = ml/kg/min; *6MWT* Six minute walk test (metres), *10MWT* 10 m walk test, *SF-36* Short Form 36, *PCS* Physical Component Summary Score, *MCS* Mental Component Summary Score, *PHQ-9* Patient Health Questionnaire

One participant in the control group was lost to follow-up

*p < 0.05. ^a^Except for VO_2_ peak where *n* = 9. ^b^*n* = 8 (Intervention), *n* = 9 (Control)

### Outcome measure completion rates

Outcome measure completion rates were high (Table 4). VO_2_ peak was calculated for 18 participants: one participant’s baseline gas exchange data was not included due to calibration issues resulting in physiologically impossible values; one participant became claustrophobic during the warm-up and was unable to tolerate the spirometry mouthpiece at baseline; follow up was not attempted. The most common reason for test termination was reaching a BORG-RPE > 17, achieved in 11 participants at baseline and eight post intervention. Other reasons for termination included inability to maintain adequate pedaling cadence and requesting to stop. Fifteen (83%) participants at baseline, and 17 (94%) post, recorded peak RER greater than 1.10. One participant was lost to follow up for walking measures and questionnaires.

### Retention and attendance

Nineteen participants completed the 12-week program (9 intervention, 10 control). One intervention group participant withdrew after seven sessions due to self-reported worsening of lower limb dystonia. The median (%; IQR) number of attended sessions was 19.5 (81%; 12, 20); and 20 (83%; 19, 22) in the intervention and control groups, respectively. Illness, including inpatient admission for co-morbid conditions, was the primary reason for non-attendance.

### Intervention delivery

Intervention group training parameters are detailed in Supplemental File 6. All intervention participants could undertake moderate-intensity (40–59% HRR) CV training. The primary mode of fitness training was upright stationary bike, followed by treadmill, recumbent bike, stepper, cross trainer, repetitive step ups or stair climbing, and arm ergometer.

Progression to the target training duration (30 min) was gradual, requiring a mean of 10 (range 6–19) sessions. The mean (SD) duration at target heart rate was 21.5 (7.9) minutes across the 12-week trial. This increased to a mean 27.8 (4.4) minutes in the final 4 weeks of the trial. The protocol required 30-min training duration before increasing intensity and as such, peak intensities achieved were relatively modest: two participants achieved 55% HRR, three 50% HRR, one 45% HRR and three remained at 40% HRR. The one participant who withdrew did not progress beyond 40% HRR.

Weekly home exercise data collection sheets, including a tick box section for participants to indicate adherence, were inadequately completed and returned. This limited accurate recording or analysis of home exercise adherence.

### Adverse events and safety

No adverse events were reported during testing or training sessions in either group. One participant presented with tachycardia before training and did not commence the session; subsequent medical clearance from their cardiologist was sought and granted to continue trial participation. One VO_2_ peak testing session was postponed (and successfully rescheduled for 1 week later) as the participant recorded a low blood sugar level on the day of testing.

### Usual care

Both groups were comparable in usual care physiotherapy attendance (median (IQR): intervention 10.5 (5, 22); control 10.5 (3, 22)). All participants were involved with at least one other allied health discipline (median 2.5, range 1, 8); with 10 participants (50%) involved with at least four disciplines including physiotherapy (Supplemental File 7).

### Cohort outcomes and effect sizes

Both groups recorded statistically significant improvements in mean VO_2_ peak (intervention 2.6 ml/kg/min, *p* < 0.02; control 3.0 ml/kg/min, *p* < 0.01) from baseline to follow-up, but there were no differences between groups. Both groups also experienced statistically significant improvements in peak watts across the study period (*p* < 0.05, Supplemental File 8). Mood and SF-36 mental component summary (MCS) score improved significantly in the control group (Table 4). See Supplemental File 9 for all SF-36 domain results. The proportion of participants with PHQ9 scores ≥10 (depressive illness) at follow-up was 1/10 (10%) and 0/9 (0%) in the intervention and control group respectively.

There were no statistically significant differences between the groups for mean change in any outcome measure (Table 5). There were no between-group differences in peak watts (control 14.0 (12.6), intervention 21.2 (12.5); mean difference [95% CI] 7.2 [− 4.6, 19.0], *p* = 0.22). Effect sizes were generally small, though the estimates were imprecise with large confidence intervals (Tables 4 and 5). The largest between-group effect size point estimates in change scores were seen for the SF-36 MCS and PHQ-9, in favour of the control group; and the 6MWT in favour of the intervention group (Table 5). Estimated sample sizes per group for a future RCT are included in Supplemental File 10.

**Table 5:** Change scores and effect sizes, mean (SD) unless otherwise specified

	Intervention (***n*** = 10)	Missing	Control (***n*** = 10)	Missing	Mean difference (95% CI)	Effect size	***P***
VO_2_ peak	2.56 (2.69)	2	2.98 (2.63)	1	−0.41 (−3.1, 2.3)	− 0.168 (− 1.4, 1.0)	0.75
6MWT	42.5 (74.7)	0	26.1 (53.8)	1	16.39 (−47.3, 80.1)	0.264 (−27.7, 28.2)	0.59
10MWT Self	0.044 (0.21)	0	0.022 (0.20)	1	0.022 (−0.18, 0.22)	0.113 (0.0, 0.2)	0.82
10MWT Fast	0.10 (0.31)	0	0.11 (0.18)	1	−0.006 (− 0.26, 0.25)	−0.041 (− 0.2, 0.1)	0.96
SF-36 PCS^a^	2.67 (6.41)	2	2.51 (11.18)	1	0.16 (−9.43, 9.75)	0.018 (−4.1, 4.2)	0.97
SF-36 MCS^a^	5.85 (9.31)	2	11.51 (14.86)	1	−5.67 (−18.70, 7.36)	−0.479 (− 6.1, 5.1)	0.37
PHQ-9	−2.8 (7.4)	0	−4.3 (3.0)	1	1.53 (−4.0, 7.1)	0.275 (−2.2, 2.7)	0.57

*CI* Confidence interval. *p* values are calculated using independent samples *t*-tests; except for the 10MWT analyses which were performed using the Mann Whitney U test. VO_2_ peak = GXT (mL/kg/min); *6MWT* Six minute walk test (metres), *10MWT* 10 m walk test (m/s), *SF-36* Short Form 36, *PCS* Physical Component Summary Score, *MCS* Mental Component Summary Score, *PHQ-9* Patient Health Questionnaire; total out of 27

One participant in the control group was lost to follow-up [remaining text correct]

## Discussion

This pilot study demonstrated the implementation of moderate-intensity CV fitness training following stroke was safe in addition to usual care, in our sample, in a hospital-outpatient rehabilitation setting. Supervised-session attendance was good and comparable to previous studies (72% (7) and 65–100% (12)), demonstrating most enrolled participants were willing to engage in fitness training. Outcome measurement was feasible, including aerobic graded exercise testing. Whilst it has been suggested that exercise testing is often terminated for non-cardiopulmonary reasons following stroke [35], the achievement of RER > 1.10 in 84% of tests shows near maximal effort was attained by most participants, with 1.0 previously suggested as reasonable criterion for maximum effort in the stroke population [27]. However, challenges were identified: lower than expected recruitment rates, and achievement of the recommended CV training dosage (30 min, 5 days/week) across the 12-week trial.

Eligibility and consent rates were low. This could be partly attributed to the usual prevalence of co-morbidities, and contraindications to peak aerobic testing, in the post-stroke population [13, 36]. With appropriate medical supervision, future pilot studies could test the safety of relaxing the inclusion criteria to peak aerobic testing, given the paradox in the prohibition of those who potentially have most to benefit from participating in cardiovascular training either in research or practice. Frequently, reasons cited for non-consent were consistent with previously identified barriers to post-stroke exercise participation: access factors (transport and inability to drive); health problems; and, stroke-related impairments [37]. Although funding of transport costs may be beneficial to facilitate recruitment, such low consent rates raise concern about patient and family perceptions of fitness training following stroke, which warrants further qualitative investigation [37]. It is noteworthy that the enrolled sample comprised a high proportion of male participants (90%), compared with the Australian stroke population (55%) [38] and our screened sample (65%). Alternative trial designs (adaptive clinical trials [39, 40], stepped wedge [41, 42]) could be considered to facilitate the feasibility of trial conduct and acquittal, as well as strategies to ensure appropriate representation of women.

This trial demonstrates a gap between guideline recommendations and actual intervention delivery. Progression of training duration was slow (mean 10 sessions/5 weeks to reach 30 min) in the recruited cohort of stroke survivors, who had very poor baseline fitness levels [13], below those previously recorded in community ambulatory stroke survivors [43]. This limited training volumes achieved (21 min mean duration at target HR) across the 12-week training period. Similarly, previous studies reporting actual dosage achieved by stroke survivors, as opposed to prescribed dose, found mean durations limited to 15 to 25 min at THR over 4 to 19-weeks [44, 45]. Of note, 88% of participants in this trial reached 30 min and the actual dosage achieved in the final 4 weeks was a mean duration (at THR) of 27.8 (4.4) minutes. This demonstrates most of the stroke survivor cohort did reach the target duration parameters but required a lengthy adaptation period.

Furthermore, intensity progression was modest (only 50% reached or exceeded 50% HRR). Following the ACSM guidelines, which recommend achievement of target duration prior to intensity progression [13], meant participants could not maintain previously achieved durations if intensity was increased. Variable intensity achievement (peak THR) has been previously reported: mean of 48–54% HRR [44]; 80% exceeded 50% HRR [45]; 30% of participants unable to progress beyond 40% HRR, whilst 70% achieved over 50% HRR [35]; however, it is unclear if these protocols required the achievement of requisite duration prior to progressing intensity. Improved reporting of adherence to aerobic training programs is needed in future trials [12, 16].

It is logical that stroke-related impairments and low cardiovascular fitness impair stroke survivors’ ability to physically achieve target training parameters. Our results suggest adherence to training frequency may also play a role: the three participants unable to progress intensity (> 40%HRR) attended less than 70% of centre-based sessions, two of which did not achieve the target 30-min session duration. The primary reason for non-attendance (illness) reflects a cohort of patients where co-morbidities and stroke sequelae limit consistent participation in fitness training, which may subsequently hinder training progression. It is unknown if poor compliance with recording the home exercise sessions corresponded to poor home exercise adherence, and thus training frequency, but this is likely. In this study, both groups received similar encouragement and training to complete their HEP, as typically done in clinical practice, and it would be useful for further research to evaluate methods of improving home exercise program adherence.

Since the commencement of this trial, exercise recommendations in stroke survivors have been revised, maintaining the aim of 20–60 min’ duration, but acknowledging that multiple short bouts of moderate intensity exercise repeated throughout the day may be better tolerated than aiming to achieve duration targets in a single session (as evidenced by our trial) [46, 47]. Interval training, including high intensity, is feasible in stroke survivors [44, 48, 49], and warrants further investigation as a method of enabling duration and intensity achievements in the post-stroke population [16].

Both groups experienced statistically significant improvements in VO_2_ peak, which were comparable to meta-analyses [7, 12, 50]. VO_2_ peak at follow-up exceeded the requirement for independent living [10] in the intervention group only, although the control group baseline VO_2_ peak was lower (non-significant). Improvements in the control group suggest aerobic benefit may be derived from lower volumes or intensities than predicted (fitness gains with intensities as low as 30% HRR have been demonstrated in the deconditioned cardiac population [51]). However, this is not supported by pooled meta-analyses which identified higher training intensity and higher baseline VO_2_ peak as the only factors significantly associated with greater improvements in aerobic capacity following stroke [16, 50].

Further research is required to understand both the minimum volume of training for effect in stroke survivors, particularly in those with very low baseline fitness; and to determine dose-response-intensity relationships, to elicit the most effective methods of restoring cardiovascular fitness in the post-stroke population, to benefit function, and reduce risk of further events [46]. Furthermore, to accurately test the target training dosage, the inclusion of an adaptation/conditioning period should be considered in future trials to meet minimum dosage requirements before proceeding to efficacy evaluation. However, the feasibility of sustained implementation of prolonged, supervised training programs in routine clinical practice is limited and highlights the need to further explore strategies to optimise adherence to the recommended frequency (5 days/week), including regular home or community-based fitness training [52]. Funding wearable activity-tracking technology devices or smartphone apps for accurate recording and analysis of home exercise adherence (including intensity and duration) could be considered in future trials [53].

The small sample size limited the estimate of intervention effect; usual in a pilot trial where the primary aim is to ascertain feasibility [21]. There were no statistically-significant between group differences and effect sizes were generally small, however it is likely the theoretical effect of moderate-intensity fitness training was underestimated as most participants were only able to reach the recommended training volume for the latter part of the trial [12]. Whilst our active control group minimized bias by providing equal exposure time, the regular extra exercise duration, albeit of a low intensity, may have elicited an improvement in fitness in these participants with low baseline fitness levels [12]. The ‘active’ control protocol was designed to mimic the prevailing model of care, however, given the modest progression of intensity in the intervention group, there was unexpectedly little separation in training intensity between groups, and this is an important issue that must be considered in the design of future adequately-powered RCTs, although as acknowledged recently by Saunders and colleagues, it is possible that engagement in exercise which is regular and progressive is the more important intervention, than prescription of precise intensity [12].

The control group did experience significant improvements in mood. However, half of the control group received clinical psychology during their rehabilitation (compared to 30% of the intervention group). Given the dynamic natural history of post-stroke depression [4], future trials should consider relevant covariates and co-interventions in adjustment of results in the primary outcome.

### Limitations

As this is a pilot study, data of effect should be considered as demonstration of feasibility only, and not indicative of true effect, for which an adequately-powered RCT would be required. Use of age-predicted maximum heart rate to calculate exercise intensity as a percent of heart rate reserve, rather than actual maximum heart rate, may contribute to challenges in intensity achievement, although no intervention participants were taking beta-blockers, which can result in lower than expected maximum heart rates, heart rate response to exercise, or both [54].

Budget limitations prevented funding for transport, dedicated staffing for enrollment and sophisticated data collection methods for home exercise adherence. This study did not aim to test feasibility of CV training in patients who were aphasic, non-ambulant or within six-weekspost-stroke. Recent research has shown early aerobic exercise (commencing at 6 days after stroke) is feasible [9]. Future studies could consider earlier implementation of intervention, particularly indicated given training adaptation periods demonstrated in this trial; however, the AVERT trial suggests that high-dose intervention in the first 24 h following stroke may not be beneficial [55].

## Conclusions

A protocolised moderate-intensity cardiovascular fitness training program was safe in stroke survivors, in addition to usual care. VO_2_ peak significantly improved in both groups, who had very low baseline fitness. There were no between group differences, however progression of training parameters was slow, subsequently limiting the overall dosage provided. A large RCT with power to make significant conclusions about the impact of moderate-intensity CV training program on the defined outcomes is now required; alternative protocols and dose-response relationships could also be trialled for feasibility to determine the most effective training method to improve functional capacity and limit the risk of stroke recurrence.

## Supplementary Information

**Additional file 1: Supplemental File 1.** Full list of inclusion and exclusion criteria. **Supplemental File 2.** Additional Intervention (Exercise) Progression Details. **Supplemental File 3.** Home Exercise Program Details. **Supplemental File 4.** VO2 Peak Graded Exercise Testing Procedure. **Supplemental File 5.** Additional detail on SF-36 and PHQ-9.**Supplemental File 6.** Training parameters achieved in centre-based sessions. **Supplemental File 7.** Other allied health intervention by group (n, %). **Supplemental File 8.** Peak Wattage Pre-Post.**Supplemental File 9.**SF-36 domains pre-post within groups for both intervention and control groups. **Supplemental File 10.** Estimated sample size calculation for future RCT.

## Data Availability

All data and materials (including Participant Information and Consent Forms) available from corresponding or senior author (on reasonable request). All authors, external and internal, had full access to all data (including statistical reports and tables) in the study and can take responsibility for the integrity of the data and the accuracy of the data analysis.

## Abbreviations

6MWT: Six minute walk test
10MWT: Ten metre walk test
ACSM: American College of Sports Medicine
CBR: Community-based rehabilitation
CONSORT: Consolidated Standards of Reporting Trials
CV: Cardiovascular
ECG: Electrocardiogram
GXT: Graded exercise test
HEP: Home exercise program
HREC: Human Research Ethics Committee
HRR: Heart rate reserve
ICC: Intraclass correlation coefficient
IQR: Inter-quartile range
MCS: Mental Component Summary score (of the SF 36)
mRS: Modified Rankin scale
NIHSS: National Institutes of Health Stroke Scale
PCS: Physical Component Summary score (of the SF 36)
PHQ-9: Patient Health Questionnaire
QOL: Quality of life
RCT: Randomized controlled trial
RER: Respiratory exchange ratio
RPE: Rating of perceived exertion
SD: Standard deviation
SF36: Short Form 36
THR: Target heart rate
VO_2_: Peak oxygen uptake
