# Supplementary material for: Feasibility and adherence to moderate intensity cardiovascular fitness training following stroke: a pilot randomized controlled trial
Source: BMC Neurol. 2021 Mar 22;21:132. doi: 10.1186/s12883-021-02052-8 (PMC7983371; doi:10.1186/s12883-021-02052-8)
Supplement: Supplementary file 1 — Additional file 1: Supplemental File 1. Full list of inclusion and exclusion criteria. Supplemental File 2. Additional Intervention (Exercise) Progression Details. Supplemental File 3. Home Exercise Program Details. Supplemental File 4. VO2 Peak Graded Exercise Testing Procedure. Supplemental File 5. Additional detail on SF-36 and PHQ-9.Supplemental File 6. Training parameters achieved in centre-based sessions. Supplemental File 7. Other allied health intervention by group (n, %). Supplemental File 8. Peak Wattage Pre-Post.Supplemental File 9.SF-36 domains pre-post within groups for both intervention and control groups. Supplemental File 10. Estimated sample size calculation for future RCT. [file 12883_2021_2052_MOESM1_ESM.docx]

**SUPPLEMENTAL MATERIALS**

**Supplemental File 1. Full list of inclusion and exclusion criteria.**

**Inclusion criteria:**

- Aged 18 years and over
- Diagnosed with stroke (ischaemic or haemorrhagic) at least six weeks and no more than 12 months prior
- Able to walk at least 100m, with or without a gait aid or supervision from another person.

**Exclusion criteria:**

- Inability to physically participate in a cycle ergometry test (unable to safely transfer on and off a stationary bike, unable to pedal)
- Documented medical restrictions to cardiovascular training in medical history
- Inability to follow two-stage commands or indicate symptoms during exercise
- Pregnancy
- Unstable angina
- Acute or complex arrhythmia
- Severe aortic valve disease or stenosis
- Severe left ventricular dysfunction
- History of unstable cardiac failure or uncontrolled hypertension
- Recent myocardial infarction requiring acute intervention (within six weeks prior to recruitment)
- Acute myocarditis or pericarditis
- Acute pulmonary embolism or infarction
- Pulmonary hypertension
- Interstitial lung disease
- Severe renal impairment
- Acute infection or fever (local or systemic)
- Uncontrolled diabetes (deemed by assessing neurology physician)
- Neurosurgery within six weeks prior to recruitment date

**Supplemental File 2. Additional Intervention (Exercise) Progression Details**

Exercise duration was progressed prior to increasing intensity, as recommended by ACSM (13). Intensity was progressed when 30 minutes’ duration was achieved, by a maximum 5% increment (Karvonen method) each session. Progression was based on individual capacity on training day and tolerance of previous sessions (heart rate recovery time, self-reported fatigue).

Selection of training mode was dictated by physiotherapy assessment of physical capacity and safety, and patient preference. Blood pressure, resting heart rate, blood sugar levels (diabetic patients) and medication changes were monitored before and after training, and training was postponed if predetermined safety parameters were not met. Heart rate was monitored with chest strap or pulse oximeter to assess whether participants reached target heart rate. BORG-Rating of Perceived Exertion (BORG-RPE) was recorded regularly (translated BORG charts utilised as required). Intervention therapists provided verbal encouragement or adjusted fitness machine levels to optimise intensity during training.

**Supplemental File 3. Home Exercise Program Details.**

Once participants were deemed safe to exercise independently, self-monitor their own heart rate or BORG-RPE scale, and recognise symptoms warranting cessation of CV training, the HEP was prescribed, to be completed 3 days per week. Participants were provided with standardised HEP instruction and recording sheets outlining duration & intensity targets (see below).

Prescribed weekly, HEP durations matched supervised session durations achieved. The predominant modes of home exercise were walking, fast repetitive sit to stand or step ups, which did not require specialised equipment. Participants utilised their own exercise bikes at home where available. Control group HEP reflected duration and exercises performed in supervised sessions. Participants were instructed not to exercise above BORG-RPE 10 (low intensity).

**Home Exercise Program Information Sheet**

You will be asked to complete a home exercise program as part of the research project. Your exercise program will be given to you each week.

If your usual physiotherapist has given you an exercise program, please continue with this as well.

- Always drink water before and after exercising
- Advise the physiotherapist if your medications have changed
- Advise the physiotherapist if you have any new medical issues

**People with diabetes**

- Make sure to eat & test blood sugar levels before exercising

**­­STOP EXERCISING IF YOU HAVE:**

- Chest pain, palpitations or angina
- New symptoms of dizziness
- Difficulty breathing

Seek medical attention

- New pain during exercise
- Nausea
- New tingling or numbness
- A fall

**EXERCISE MONITORING**

You will be asked to monitor how hard you are exercising. The physiotherapist will teach you about how to monitor this.

**Rating of Exertion** (circle)- Aim to exercise at level circled. Record level on exercise sheet.

**Rest** if rating is more than __________


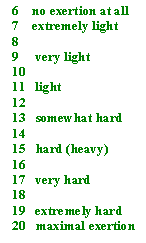


If you have any questions, please call _______________

**Home exercise program recording sheet**

**Week Participant to complete ____________home exercise sessions this week**

***Therapist to complete***

**Exercise Type recommended:**

**Recommended time:**

**Target Heart Rate:**__________

Rest if above:

**Rating of Perceived Exertion:**

Rest if above:

***Session 3* Date:**____________

**Home exercise done?**

**Y** € **N** €

**If No – why?**_________________

**Total exercise time:** ___________

| **Time** | **Rating of Exertion** | **Heart Rate** |
| --- | --- | --- |
| 5 minutes |  |  |
| 10 mins |  |  |
| 15 mins |  |  |
| 20 mins |  |  |
| 25 mins |  |  |
| 30 mins |  |  |

***Session 2* Date:**____________

**Home exercise done?**

**Y** € **N** €

**If No – why?**_________________

**Total exercise time:** ___________

| **Time** | **Rating of Exertion** | **Heart Rate** |
| --- | --- | --- |
| 5 minutes |  |  |
| 10 mins |  |  |
| 15 mins |  |  |
| 20 mins |  |  |
| 25 mins |  |  |
| 30 mins |  |  |

***Session 1* Date:**____________

**Home exercise done?**

**Y** € **N** €

**If No – why?**_________________

**Total exercise time:** ___________

| **Time** | **Rating of Exertion** | **Heart Rate** |
| --- | --- | --- |
| 5 minutes |  |  |
| 10 mins |  |  |
| 15 mins |  |  |
| 20 mins |  |  |
| 25 mins |  |  |
| 30 mins |  |  |

**omeHome**

**Supplemental File 4. VO_2_ Peak Graded Exercise Testing Procedure.**

Testing was conducted with standardised instructions using a ramp protocol. Cycle ergometry was used to reduce the influence of balance problems or gait impairments on fitness testing (27). Participants with severe upper limb impairment were not excluded (only used non-affected upper limb on ergometer handlebar). The respiratory exchange ratio (RER) [the ratio between the amount of carbon dioxide (CO_2_) produced in metabolism and oxygen (O_2_) used], was recorded to assess the level of physical exertion during the GXT (13), where peak RER > 1.10 is considered to be an indication of the subjects’ maximal effort.

*Setting and safety*

The GXT was conducted on-site by an accredited exercise physiologist experienced in exercise testing, with two clinical personnel always in attendance. Emergency equipment and medical physicians were on-site and immediately available to provide medical assessment or intervention if required.

*Pre-testing procedure*

Body weight, ECG, blood sugar levels and blood pressure were assessed prior to testing. Testing proceeded only if the following contraindications were not met:

- Significant change in the resting ECG suggesting significant ischemia or recent myocardial infarction;
- Severe hypertension (SBP>200 and/or DBP>110 mmHg at rest;
- Resting tachycardia (HR>100 bpm);
- BSL <4 or > 16mmol

*Testing Procedure*

Pre-screen and instructions

The test was a sign- or symptom-limited graded (incremental) exercise test conducted on a stationary cycle ergometer. Participants were screened prior to recruitment to ensure adequate ability to cycle stationary bike and transfer safely.

Participants were provided with standardized instructions to pedal at a cadence that they felt comfortable with (50-70 revolutions per minute (RPM)) and indicate a rating of perceived exertion (RPE - BORG scale 7-20) by pointing to a score card each minute.

Test protocol

Participants rested for 2 minutes after which they began cycling and the workload resistance was increased according to a ramp protocol, commencing at 0 watts, then progressed by 20 watts (up to 60 watts in males), then 10 watts, every minute. In females, the resistance progression was 10 watts every minute after the 20 watts workload.

Monitoring and feedback

Oxygen consumption and the respiratory exchange ratio (RER) were recorded throughout each test using open circuit spirometry via a portable metabolic system (Medgraphics, Ultima CPX, Saint Paul, MN). Heart rate (HR) was recorded continuously via 12-lead electrocardiogram (ECG) device (Mortara, X-Scribe II, Milwaulkee, WI). Blood pressure was not routinely measured during GXT due to the lack of availability of an accurate measurement tool. Feedback was provided regarding participant’s maintenance of RPM. Participants were progressed to a maximum RPE of 17.

*Post Testing*

Blood pressure and BSL were monitored on completion of testing.

**Indications for termination of exercise testing before RPE 17**

- Participant is fatigued, exhausted or asks to stop
- Pain (of musculoskeletal origin)
- Apprehension; mental confusion or change in conscious state
- Angina or chest pain;
- Wheezing, leg cramps, claudication;
- Drop in SBP>10mmHg despite an increase in work load;
- Increasing nervous system symptoms (ataxia, dizziness, or near syncope);
- Signs of poor perfusion (cyanosis or pallor - lack of colour; unnatural paleness);
- Sustained loss of ECG signal, and;
- Sustained ventricular tachycardia (SVT)
- ST-elevation (+ 1.0 mm) in leads without diagnostic Q-waves (other than V1 or aVR)
- ST or QRS changes (such as ST depression> 2mm horizontal or down sloping);
- Arrhythmias other than SVT (sustained ventricular tachycardia), PVC (premature ventricular contraction), supraventricular tachycardia, heart block or brady arrhythmias;
- Development of bundle-branch block;
- Hypertensive response: SBP>250mmHg and/or DBP > 115 mmHg.

**Supplemental File 5. Additional detail on SF-36 and PHQ-9.**

The Short Form-36 (SF-36)*,* Australian version, Version 2 (31, 32) is a generic health-related quality of life measure with 8 domains and two component summary scores (physical and mental). Normed T-scores (population mean is 50 and standard deviation 10) are presented (31). The SF-36 has acceptable to good reliability and satisfactory internal consistency in stroke survivors. The vitality subscale is considered a valid and reliable measurement of post-stroke fatigue and the physical functioning (PF) subscale is a valid assessment of long-term physical health in stroke survivors. Other domains should be interpreted with caution due to psychometric issues.

The English-language version of the Patient Health Questionnaire (PHQ-9), a 9-item scale (total scores range from 0-27) is recommended as a screening tool for post stroke depression (4). THE PHQ-9 discriminates equally well regardless of age, gender or ethnicity and has a sensitivity of 0.86 and specificity of 0.79. If depression was indicated by the PHQ-9 (score≥10), participants were informed and offered referral to clinical psychology.

**Supplemental File 6. Training parameters achieved in centre-based sessions.**

Duration progression was primarily limited by subjective reports of fatigue (participant requesting to stop) or inability to maintain target heart rate over a longer training duration (even if training bouts were utilised).

After participants achieved 30 minutes duration, therapists aimed to progress intensity (target heart rate individually calculated using the Karvonen method) every session. Intensity progression was limited by participants’ inability to achieve previously established training duration, or BORG RPE exceeding the upper threshold of moderate intensity, if target heart rate (intensity) was increased.

| **Intervention Participant** | **Total number of sessions attended** | **Maximum training duration at target HR achieved (minutes)** | **Number of sessions to progress to maximum training duration** | **Mean training duration at target HR (minutes)** | **Peak Intensity (%HRR)** |
| --- | --- | --- | --- | --- | --- |
| 1 | 20 | 30 | 12 | 21.95 | 55 |
| 2 | 24 | 30 | 19 | 20.3 | 50 |
| 3 | 20 | 30 | 9 | 24.2 | 45 |
| 4 | 20 | 30 | 12 | 23 | 50 |
| 5 | 12 | 30 | 10 | 15.1 | 40 |
| 6 | 19 | 30 | 7 | 24.5 | 50 |
| 7 | 7 (withdrew) | 30 | 4 | 22.8 | 40 |
| 8 | 10 | 27 | 9 | 17.2 | 40 |
| 9 | 16 | 23 | 13 | 15.4 | 40 |
| 10 | 20 | 30 | 5 | 26.2 | 55 |

**Supplemental File 7. Other allied health intervention by group (n, %).**

| **PROFESSION** | **COHORT** | **INTERVENTION** | **CONTROL** |
| --- | --- | --- | --- |
| Occupational Therapy | 17 (85%) | 10 (100%) | 7 (70%) |
| Clinical Psychology | 8 (40%) | 3 (30%) | 5 (50%) |
| Speech Pathology | 11 (55%) | 9 (90%) | 2 (20%) |
| Dietetics | 4 (20%) | 2 (20%) | 2 (20%) |
| Social Work | 8 (40%) | 4 (40%) | 4 (40%) |
| Podiatry | 1 (5%) | 0 (0%) | 1 (10%) |
| Nursing | 2 (10%) | 1 (10%) | 1 (10%) |
| Neuropsychology | 2 (10%) | 1 (10%) | 1 (10%) |
| Rehabilitation Consultant | 10 (50%) | 5 (50%) | 5 (50%) |
| Community Integration | 4 (20%) | 3 (30%) | 1 (10%) |

**Supplemental File 8. Peak Wattage Pre-Post**

|  | **Control**  **(n = 10)** | **Intervention**  **(n = 10)** |
| --- | --- | --- |
| Pre  Post  MD (95%) CI  *p* | 72.0 (13.2)  86.0 (22.7)  14.0 (5.0, 23.0)  0.007 | 102.8 (22.2)  124.0 (29.1)  21.2 (12.2, 30.2)  < 0.001 |

**Supplemental File 9. SF-36 domains pre-post within groups for both intervention and control groups.**

|  | **Baseline Intervention**  **(n = 10)** | **Follow-up Intervention**  **(n = 10)** | **MD**  **(95% CI)**  **(n = 10)** |  | **Baseline Control**  **(n = 10)***** | **Follow-up Control**  **(n = 10)***** | **MD**  **(95% CI)**  **(n = 10)***** |  |
| --- | --- | --- | --- | --- | --- | --- | --- | --- |
| PF  RP  BP  GH  V  SF  RE  MH | 40.1 (8.4)  36.6 (9.0)  49.3 (14.0)  44.3 (12.2)  42.4 (10.5)**  39.4 (11.9)***  43.4 (13.1)  46.1 (12.8) | 42.6 (11.3)  40.5 (12.2)  47.4 (13.1)  47.6 (12.9)  52.5 (9.8)**  50.6 (7.9)***  39.6 (19.9)  47.9 (15.6) | 2.5 (-4.7, 9.7)  4.0 (-5.1, 13.1)  -1.8 (-15.6, 11.9)  3.3 (-1.2, 7.7)  10.1 (4.5, 15.8)  11.2 (4.4, 18.0)  -3.8 (-16.0, 8.4)  1.8 (-7.2, 10.8) |  | 30.3 (11.1)  28.6 (8.9)  45.2 (12.8)  42.6 (7.8)  43.7 (9.5)  31.9 (14.3)  25.2 (22.7)  38.2 (16.2) | 35.9 (10.8)  31.6 (12.3)  51.0 (9.4)  45.8 (10.3)  49.3 (8.9)  45.0 (9.3)  38.4 (21.3)  45.7 (9.6) | 5.6 (-3.0, 14.2)  3.0 (-6.3, 12.3)  5.8 (-0.3, 12.0)  3.1 (-1.9, 8.2)  5.7 (-1.1, 12.5)  12.1 (2.3, 23.8)  13.2 (0.2, 26.3)  7.5 (-1.7, 16.7) |  |

CI, confidence interval. SF-36 = Short Form 36; PF = Physical Functioning; RP = Role Physical; BP = Bodily Pain; GH = General Health; V = Vitality; SF = Social Functioning; RE = Role Emotional; MH = Mental Health.

*p < 0.05. **n = 8 able to be analysed (missing data this domain/group); ***n = 9 able to be analysed (missing data this domain/group).

**Supplemental File 10. Estimated sample size calculation for future RCT**

Estimated sample sizes per group for a future RCT, using a two-sided t-test for difference between two independent group change scores, using an alpha of 0.05, a power of 80% and an allocation ratio of 1/1 and the 6MWT as the primary outcome (normative data, feasible) were 227 in each group (total n = 454, not including recruitment for attrition). To detect a difference with 90% power (other assumptions unchanged), sample sizes were 303 in each group (total n = 606, not including attrition).
